# Capabilities, Opportunities, and Motivation: Exploring Fitness Program Experiences of Adults with Intellectual and Developmental Disabilities

**DOI:** 10.3390/ijerph20105771

**Published:** 2023-05-09

**Authors:** Melissa N. Savage, Andrew M. Colombo-Dougovito

**Affiliations:** 1 Department of Educational Psychology, University of North Texas, Denton, TX 76203, USA; 2 Department of Kinesiology, Health Promotion and Recreation, University of North Texas, Denton, TX 76203, USA

**Keywords:** physical activity, intellectual and developmental disabilities, adults, qualitative methods, COM-B

## Abstract

Although there are many benefits to regular engagement in physical activity, adults with intellectual and developmental disabilities often do not engage in or near the recommended amount of physical activity. Barriers, such as lack of perceived capability, accessible environments, transportation, social support, and or knowledgeable support staff, may limit participation in physical activity. The present study used qualitative methods to explore the experiences of adults with intellectual and developmental disabilities enrolled in a fitness program. We used field observations and photo-elicited semi-structured interviews to explore what capabilities, opportunities, and motivations facilitate or hinder engagement in fitness classes and their experiences in the program. We used the COM-B model to deductively interpret and analyze the data through thematic analysis. Major themes were identified around types of support and preferences for physical over sedentary activities. Instructor, client, and family support were identified as important in facilitating interest, engagement, and skill. Dependency on others for financial and transportation support was also reported as important for participants to access the fitness program. This study provides valuable insight into the interactions and experiences of adults with intellectual and developmental disabilities, including what keeps them engaged in a fitness program as it relates to capabilities, opportunities, and motivation.

## 1. Introduction

Physical fitness includes characteristics enabling people to perform physical activity with health-related components such as cardiorespiratory endurance, muscle strength, muscle endurance, flexibility, and body composition [1]. Across the years, these components continue to be key components in health-related physical fitness [2,3]. While physical fitness is an important part of a healthy lifestyle, adults with intellectual and developmental disabilities (IDDs) often do not engage in recommended levels of physical fitness, and their levels of engagement are lower compared with adults without IDDs [4]. IDDs are disabilities that are typically present at birth and uniquely affect the trajectory of an individual’s physical, intellectual, and/or emotional development. Intellectual disability starts at any time before a child turns 18 and is characterized by difficulties with both intellectual functioning (i.e., the ability to learn, reason, and problem-solve) and adapted behavior (i.e., everyday social and life skills). Developmental disability is a broader category of often lifelong disability that can be intellectual, physical, or both; likewise, developmental disabilities present themselves during a child’s growth from infancy through adolescence.

Due to lower levels of physical activity, individuals with IDDs are at an increased risk for secondary conditions such as heart disease, diabetes, and obesity [5,6], and psychosocial conditions such as anxiety and depression [7,8]. Lowering the risk for these conditions can be challenging due to barriers to physical activity for individuals with IDDs. A number of factors may contribute to decreased levels of physical activity such as physical limitations and access to accessible environments [9], a lack of social support [10], a lack of knowledge at both an individual level (e.g., not knowing where to go or how to exercise) and at the professional level (e.g., limited understanding of how to make adaptations and fitness professionals’ lack of preparation to work with individuals with disabilities [9]), and challenges with transportation [11].

However, when individuals with IDDs participate in exercise programs, research has consistently indicated improvements in physical fitness levels and health profiles [12,13,14]. Researchers have presented moderate–strong evidence that physical activity significantly improves balance, muscle strength, and quality of life in persons with IDDs [15]. Individuals with IDDs should have the opportunity to participate in fitness programs offered in their communities if they choose. For this to happen, adults with IDDs need to be and feel included and capable, which may increase motivation to engage in more exercise [16,17]. In addition, they should have opportunities to be included and engage in various environments to determine which ones work best for them (e.g., regarding facility amenities, cost, location, and atmosphere). Social support from family, friends, and caregivers is also commonly reported to support sustained physical activity for adults with IDDs [18].

### Aims and Objectives

The purpose of this study was to explore fitness class experiences in adults with IDDs as they relate to capabilities, opportunities, and motivation. Gaining a better understanding from adults with IDDs engaged in fitness programs may better inform policy and practices as well as the training of fitness staff as the field moves forward. This study addressed the following research question: (1) What capabilities, opportunities, and motivations facilitate or hinder engagement in fitness classes?

## 2. Materials and Methods

### 2.1. Framework

**Figure 1:**
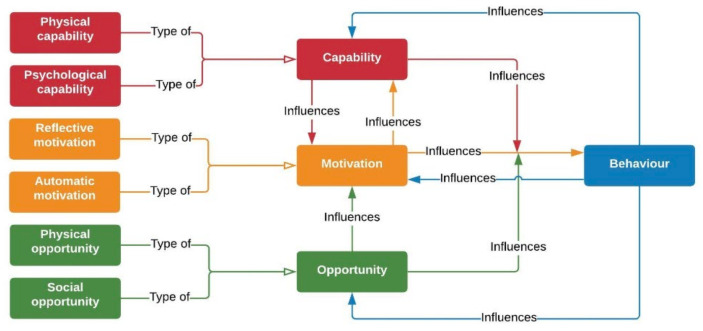
The COM-B model of behavior [17].

The Capability, Opportunity, Motivation–Behavior (COM–B) model provides a broad framework for understanding behavior. According to the COM-B model [19], for behavior change to occur, an individual must (a) be able to perform the behaviors (psychological and physical capabilities), (b) have opportunities to engage in the behaviors (physical and social opportunities), and (c) want or need to engage in the behaviors at that moment (reflective and automatic motivation). Participation in regular physical activity could significantly improve capabilities, motivation, and opportunities for physical activity, which can lead to better health outcomes for adults with IDDs. Michie et al. [19] define capabilities as “the individual’s psychological and physical capacity to engage in the activity concerned. It includes having the necessary knowledge and skills”. Opportunity is defined as “all the factors that lie outside the individual that make the behavior possible or prompt it” [19] and motivation is defined as “all those brain processes that energize and direct behavior, not just goals and conscious decision-making” [19]. Within this model, capability and opportunity often influence motivation to engage in a behavior (refer to Figure 1; [17]).

### 2.2. Engaging Adults with IDDs in Research

To best support individuals with IDDs in their fitness pursuits, we need to hear from individuals directly to learn what facilitates and hinders their engagement, motivates them to continue engagement, and what, if any, support they might need during their exercise journeys. Traditionally, research has been conducted on and about individuals with IDDs with few studies employing the perspectives of individuals with IDDs themselves [20]. Challenges researchers have faced when interviewing clients with IDDs include responses that are short, acquiescent (i.e., with a disposition to respond “yes” regardless of the question), and inconsistent [21,22]. Suggestions from the field when planning for and interviewing individuals with IDDs include (a) using simple and short questions [21]; (b) allowing sufficient time for processing, which includes planning for longer interview times and providing a wait time after asking questions [23]; (c) selecting a location where the interviewee feels safe; (d) repeating the interviewee answers to confirm their responses; and (e) asking follow-up questions or using alternative phrasing to allow interviewees to expand on their responses [20]. Using pictures to support voice may also be an approach that can strengthen the quality of interview responses from individuals with IDDs [24].

### 2.3. Research Design

We used qualitative methods to explore patterns across multiple data sources to know more about participants’ experiences, knowledge, and views about their fitness classes. The COM-B model informed the study design, data collection, and analysis. All qualitative data were transcribed and coded thematically [25]. The first author (M.N.S.) is a faculty member in special education, has a doctorate in special education, has experience collecting qualitative data, and has extensive experience working with adults with IDDs. The second author (A.M.C.-D.) is a faculty member in sport pedagogy and motor behavior, who has a doctorate in kinesiology with a focus on adapted physical education and extensive experience with qualitative methods. This study was approved by the University Institutional Review Board (IRB-19-536).

### 2.4. Sampling and Recruitment

We used purposive sampling to recruit participants who were attending a postsecondary education program for adults with IDDs located in the Southern United States. This postsecondary education program for adults with IDDs included a fitness program as part of the programming. This gave the research team access to individuals who were best suited to answer our research question. Clients in this postsecondary education program had to be 18 and have an IDD diagnosis that met the eligibility criteria in the Diagnostic and Statistical Manual of Mental Disorders, Fifth Edition, to attend the program. For this study, IDD is used to describe situations in which intellectual disability and other developmental disabilities are present.

We did not require a certain number of participants but kept recruitment open until no new information emerged related to the COM-B model. Potential participants were approached via email. The study information was sent to all clients who attended the fitness program affiliated with the postsecondary education program. Participants could participate if they (1) were 18 years or older, (2) had a documented diagnosis of an intellectual or developmental disability, and (3) participated for at least 1 year in their enrolled fitness program.

### 2.5. Participants

A total of 11 participants with IDDs between the ages of 27 and 50 participated in this study. All participants were able to follow verbal instructions, took photos with verbal and text reminders, and responded to interview questions independently. See Table 1 for demographic information. All the demographic information was self-reported by the participants or legally authorized representatives (i.e., guardians). We used documentation from the postsecondary education program to confirm IDD and age (i.e., no additional assessments were conducted by the research team to determine eligibility). Participation in the fitness program for at least one year was confirmed by the postsecondary education program’s fitness director. There were no discrepancies between the self-reported demographics and demographics confirmed by members of the research team.

### 2.6. Data Collection

Data collection phases included field observations, participants taking photos, and semi-structured interviews. No relationships were established between research team members and participants prior to the data collection. M.N.S. led the data collection across all phases.

#### 2.6.1. Field Observations

Field observations occurred first and were video-recorded to capture participant engagement during fitness classes and interactions with fitness instructors and clients (i.e., peers in the same fitness class). Field observations were used to confirm and expand upon experiences described during interviews to address previously described research challenges when interviewing individuals with IDDs, which include, short, acquiescent, and inconsistent responses [21,22]. Field observations occurred first so participants were familiar with the researcher’s presence before sitting down for their interview. In addition to the lead author video recording field sessions and taking field notes, a doctoral student in research statistics and measurement, who had training in qualitative research methods, took field notes independently from the lead author. We conducted field observations across six fitness classes. Participants were not all in the same fitness class (six participants were in one class while the remaining five were in the same class type that met at a different time and day). Each participant was included in field observations across at least three different days. Field observations lasted for sixty minutes for each observation.

#### 2.6.2. Photos for Interviews

After field observations were finished, participants were given verbal instructions for taking photos as well as visual support that included written and pictorial instructions. They were asked to take photos of people and things they felt were important related to “exercise”. Participants were told they could take photos of things they liked about exercise and/or things they did not like about exercise in any setting; additionally, participants were informed that they could choose which, if any, of their photos to share with the research team. During this process, participants were asked if they wanted to use their own phones to take photos or if they wanted disposable cameras; all participants chose to use their own phones to take photos. The purpose of the photos was for participants to have support documents available during the individual interviews to share with the researchers (i.e., there were specific photo-elicited interview questions, and the photos used were photos taken by participants). Each participant demonstrated that they knew how to take photos during the consent process. After instructions were provided, participants were given 1–2 weeks to take photos. Their instructor at the fitness center gave verbal reminders 3–4 times per week to take photos.

#### 2.6.3. Semi-Structured Interviews

While no relationships were established prior to the beginning of the data collection, interviews occurred after field observations. Participants were familiar with the lead author who video-recorded field observations and led the face-to-face interviews. Interview protocols were developed through COM-B constructs, and additional questions were finalized after field observations, including photo-elicited questions. See Table 2 for examples of interview questions developed through COM-B constructs. Before the interviews, protocols were sent to three independent experts in the field to elicit feedback. Minor edits were made to the interview questions before engaging in the interviews. M.N.S. used a list of interview questions to guide the conversation. The questions asked were short, used simple language, and were rephrased when needed to increase understanding [21,23]. The main questions often had follow-up questions ready to support participants in expanding on the topic. Participant responses were repeated back to participants for clarification. Interviews lasted for an average of 23 min ranging from 20 to 27 min. Each interview was audio-recorded and transcribed verbatim into an electronic file.

### 2.7. Data Analysis

We followed Braun and Clark’s [25] six-phase, reflexive thematic analysis process including data familiarization, generating initial codes, searching for themes, reviewing themes, defining and naming themes, and producing reports. We used the COM-B model to deductively interpret and analyze the data. After M.N.S. immersed themselves in the data to increase familiarization, data sources were iteratively coded, with codes developed or discarded as coding continued. Next, M.N.S. grouped the coded data into themes based on the COM-B constructs and developed a thematic map. Then, we conducted an analysis of each theme. At the end, we selected examples that accurately represented the generated themes and that also related to our research questions and the literature. Differences in coding were settled through a discussion between the M.N.S. and a doctoral student (i.e., a critical friend), encouraging reflexive practice by exploring multiple and alternative explanations of the interpreted data [26].

## 3. Results

The themes generated described what adults with IDDs valued in fitness classes and their experiences related to capabilities, opportunities, and motivation. See Table 3 for a description of the COM-B components, themes identified, and data presentation with illustrative extracts and corresponding analysis across the field observations and interviews.

### 3.1. Capability

*I can do it, it may be a little different, but I can do something (P01)*. In the present study, distinct themes emerged related to physical and psychological capability with barriers to physical activity including *limited coordination* and *articulation of health knowledge* and facilitators of physical activity including *instructor support*, *client support*, and *modifications*.

Participants felt least comfortable performing balance activities. Participants described difficulty with balance and coordination, as well as fear of falling. *I need to get my knees higher and need to work on my balancing more, working on my balancing and coordination too because that’s a main skill I need to work on (P05). I really am not good with balancing, kinda hard. I have a hard time since I was a baby, standing up. I like doing it but need to work on it (P09).* All participants stated that they were aware of how important exercise was as part of being healthy. Examples included *I need exercise to help lose weight (P11)* and *I exercise and I’m happy (P03).* However, most participants were not able to expand their reasoning and knowledge beyond stating that exercise was good for people.

While participants indicated some exercises were difficult, all participants reported they were good at exercise and occasionally needed modifications. Most participants stated they felt most confident when performing total body resistance exercises (TRXs). TRX is a form of suspension training that uses bodyweight exercises to develop strength, balance, flexibility, and core stability together.

Interviewer: *When you come to fitness class, what are you most excited about?*

P06: *The TRX.*

Interviewer: *The TRX gets you the most happy and excited to come.*

P06: *Yes*. (P06 got up and demonstrated the exercise while pretending to have TRX bands in their hands).

Participants stated that their instructor was very helpful in helping them engage in exercises. They felt he was a good instructor and stated they liked how he described an activity, demonstrated how to perform it, and then gave them enough turns to practice it while he helped them with their form or with modifications if necessary. They also reported their instructor’s feedback and praise were both helpful in helping them feel capable. Field observations supported participants’ physical capabilities to perform exercises with and without exercise equipment. Their instructor provided continuous feedback related to their exercise form. During observations, the instructor also provided checks for understanding (e.g., show me), additional explanations (e.g., this exercise helps your back get stronger), and encouraged participants to ask questions for clarification (e.g., do you need me to tell you that again in a different way?). Commonly observed modifications for participants included performing push-ups on the wall or from their knees in place of traditional push-ups, using a chair to prevent participants from squatting too low, holding participants’ hands during exercises, and frequent breaks.

### 3.2. Opportunity

**Figure 2:**
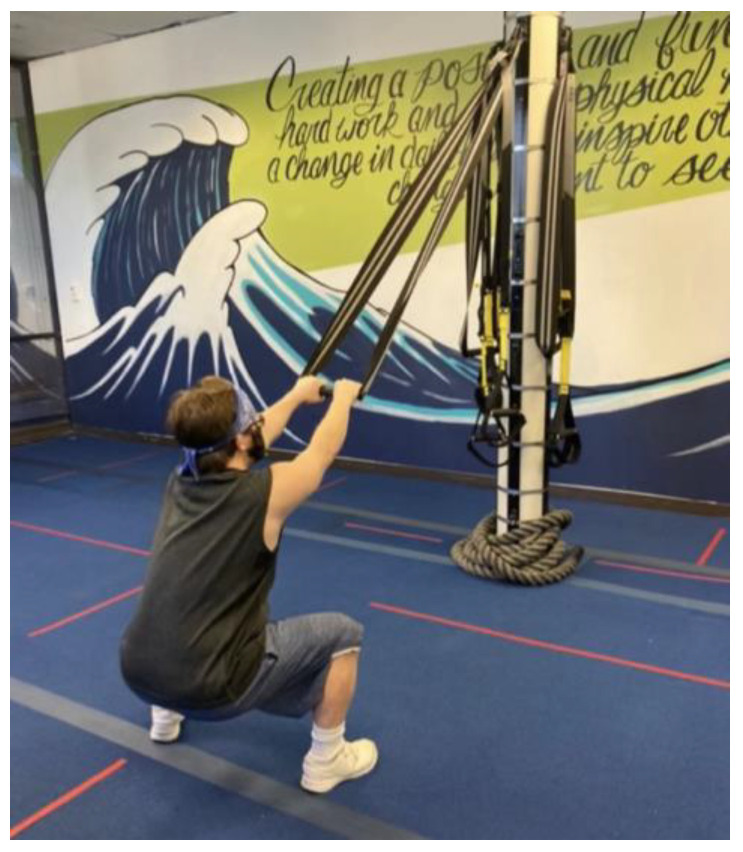
Picture participant shared during photo-elicited interview question.

In the present study, distinct themes emerged related to physical opportunity and social opportunity with barriers to physical activity including *resource dependence* and facilitators including *facility*, *instructor*, *client*, *community,* and *family support*. Many participants shared that they relied on their caregivers to bring them to the fitness classes every week and support them financially in enrolling in the fitness classes. *I don’t drive, that’s part of my Down Syndrome…my parents usually drive me everywhere (P05).* Participants described that without that support, they would not be able to go to fitness classes. Facility and family support are often related to the environmental system, including time and resources. When asked to share photos, many participants showed a photo of TRX bands (see Figure 2). This led to a discussion on how they used the bands and what they enjoyed about them. Some participants indicated social support from community members and family as well. For example, one caregiver set up a gym in their garage so they could practice exercises together outside of fitness classes, and other community members went on walks and/or bike rides with participants outside of fitness classes.

Interviewer: *Do you ever exercise at home or in other places?*

P02: *Yes, I usually just take my dog for a walk every night and ride my bike cause I got a bike too.*

Interviewer: *And, you ride your bike.*

P02: *Yes.*

Interviewer: *When you do those things do you do it on your own or does anyone go with you?*

P02: *I um, I go with my neighbor because my neighbor doesn’t want to ride bikes by herself and she wants me to go with her every time.*

Interviewer: *Yeah, that’s fantastic. Do you enjoy doing that?*

Participant 02: *Yes.*

Interview: *Out of all the places you exercise… it sounds like you exercise here, take your dog for walks, and ride your bike… where is your favorite place to exercise?*

P02: *At home.*

Interviewer: *Home is your favorite.*

P02: *Yeah. Sometimes my dad works out with me because he um, puts stuff outside and we exercise in the garage.*

Instructor and client support were frequently observed during field observations, and all participants discussed how much they liked their instructor and how their instructor made them feel good about themselves, gave them opportunities to practice, and gave them lots of praise. Many participants stated the instructor and other clients were their friends. *[Instructor name] is my friend (P05), [Instructor name] is my best friend (P03). I walk with my friend in class, we love TRX (P02).* They worked out together, talked together, and many participants shared that they spend time with one another outside of fitness classes. *We go on the bus together (P09). Me and P02 we are friends, she is funny (P01).* When asked to share a favorite picture, most pictures included themselves along with another client in the class working out together or a picture of themselves with the instructor.

Interviewer: *How was your walk?*

P01: *Good.*

Interviewer: *Do you want to tell me anything about your walk?*

P01: *No.*

Interviewer: *Let’s look at the pictures you took. You can share any pictures you want to share. Which ones do you want to share?*

P01: *[Giggles and shows interviewer picture of her taking a walk with her fitness class]. I took a selfie.*

Interviewer: *What were you doing in this picture?*

P01: *Walking. It makes me happy to walk. This is me and (insert instructor name) on the walk. I just took this one [participant smiles and laughs].*

Interviewer: *Does (insert instructor name) make it easier or harder for you to exercise?*

P01: *Easier.*

Interviewer: *What does he do that helps make it feel easy?*

P01: *Walks by my side.*

### 3.3. Motivation

In the present study, distinct themes emerged related to reflexive and automatic motivation with facilitators of physical activity including *instructor support*, *client support*, *family support*, and *preference for active activities*. No themes were identified for barriers to physical activity related to reflexive and automatic motivation. During one of the interview questions, participants were asked to rank a list of 12 activities from their favorite to least favorite and talk about a couple of activities on the list. The list included six active activities (i.e., lifting weights, individual sports games, swimming, going for a walk, team sports games, and running) and six sedentary activities (i.e., watching television, playing on the computer/tablet/phone, listening to music, playing board games, reading, and playing video games). Two-thirds of participants identified an active activity as their favorite of the twelve, and most participants (eighty-two percent) favored active activities over sedentary activities for their top six activities (i.e., four or more of their top six activities were active activities).

Several participants shared that they did not receive tangible incentives (e.g., food or tokens) for going to fitness classes. They identified exercises that were easy for them and ones that were harder. For harder exercises, participants indicated they felt supported by their instructor and other clients. Field observations supported the statements participants made in interviews. For example, a participant who indicated balance activities were a little hard and not as motivating shouted, *Oh no (P08)* and sat on the gym floor when the instructor asked them to balance on one foot. The instructor came over, helped them up, and held their hands during the first round of the balance activity. Then, they held one hand in the next round of the activity and then, for the last round, let the client try it by themselves. Another client came over and gave them a high-five after the activity. Many participants stated that their instructor, family, and other clients were helpful and gave them lots of praise; participants stated they really enjoyed engaging in fitness classes.

Interviewer: *What do you like most about the classes here?*

P04: *I love everything.*

Interviewer: *You love everything.*

P04: *Yeah.*

Interviewer: *When you walk in, what are you most excited about if you could only pick one thing?*

P04: *My favorite is [instructor name] here.*

Interviewer: *What about [instructor name]?*

P04: *[Instructor name] is my favorite thing here.*

Interviewer: *What does [instructor name] do that makes you feel great about exercise?*

P04: *He got me famous. Yes.*

Interviewer: *What does that mean?*

P04: *He got me famous. [Mumbled]. I worked so hard, so…so… student of the month. I got it twice.*

Interviewer: *Nice! How about this. If you could change anything about fitness classes, if you could change something, what would you change?*

P04: *I never change it I still love it. I love everything.*

## 4. Discussion

The present study explored fitness class experiences in adults with IDDs as they relate to capabilities, opportunities, and motivation. Qualitative details were gathered from adults with IDDs, with field observation and field notes as data sources that supported the interview data from the adults with IDDs. As described previously, we were interested in what capabilities, opportunities, and motivations may facilitate or hinder engagement in fitness classes as adults with IDDs describe their experiences and interest in those classes.

### 4.1. Barriers and Facilitators to Capability, Opportunities, and Motivation

The findings highlight the complexity of need for adults with IDDs to engage in fitness programs. The challenges participants reported are consistent with the literature for adults with IDDs related to balance activities [27,28], modifications [29], and relying on others for transportation [11]. In this study, transportation was mentioned most often as a barrier to independent participation (e.g., many participants relied on a caregiver to bring them to fitness classes). Without that support, they stated they would not be able to attend fitness classes. When adults with IDDs and their caregivers are looking to join a fitness center, they might consider fitness centers that are accessible via public transportation when possible. This could provide multiple options for adults with IDDs to access the facility and not rely solely on their caregivers for support.

While participants discussed some challenges, participant perspectives and experiences in this study were overwhelmingly positive regarding their capabilities, opportunities provided, and motivation to engage in exercise. Participants reported that they believed in themselves, had the ability to exercise, and preferred active activities, which is inconsistent with previous findings (e.g., [30,31]). Participants with IDs in the studies by McDermott et al. [30] and Shimmell et al. [31] reported a lack of confidence, low self-image in physical activity engagement, and a preference for sedentary activities. With the level of instructor support observed and described by participants in this study, the fitness instructor may play a key role in supporting and building self-efficacy for physical activity engagement in individuals with IDDs. The instructor was also observed providing varied activity opportunities throughout fitness classes (e.g., walking, lifting weights, running, and group sports games), increasing exposure to different activities. Exposure to and practice in different active activities may also support a preference for active activities [32]. Participants felt supported by their instructor, peers, and families and looked forward to their fitness classes each week. These findings extend the literature suggesting the importance of establishing positive relationships with instructors, peers, and family members [33]. In the study by McDermott et al. [30], peer support was a primary reason for participating in physical activity for individuals with IDDs. Additionally, previous researchers have generally viewed caregivers as a “gatekeeper” role regarding their child’s physical activity, implying they have the power to choose whether to promote physical activity and to what extent [33].

Throughout the field observations and interviews, different COM-B constructs appeared to be influencing one another. For example, belief in their capabilities as well as opportunities were both motivations for engagement in fitness classes. *I like that I can do it, it makes me want to come more (P07). [Instructor name] is my favorite, so I come twice week now, I wish I come more (P01).* This is consistent with a quote from the study by West and Michie [17] when describing the Com-B model: “the greater the capability and opportunity the more likely a behavior is to occur because the more often the ‘gates’ will be open when the motivation is present.” Instructor and client support were also themes identified across each of the COM-B components. Both appeared to be important to participants and, based on the field observations and interviews, the instructor and clients were essential for creating a supportive and motivating environment, providing opportunities, and building capabilities to facilitate physical activity for adults with IDDs.

### 4.2. Involving Adults with IDDs in Research: Capturing Experiences

The interview practices implemented by M.N.S. followed practices used previously by researchers interviewing individuals with IDDs [23]. We asked short, simple questions by breaking down a question into smaller questions (see Table 2 for some examples). We provided a wait time of five seconds or longer depending on participant need [23] and interviewed participants in a familiar location, at their fitness center. We frequently repeated participants’ answers to confirm their responses and asked follow-up questions, so participants had the opportunity to expand on their answers. Follow-up questions were also used to confirm original responses. For example, when participant four stated *I love everything (P04)*, we followed up to obtain some specific examples. We also asked if he would change anything about the fitness class, and he stayed consistent with his original response. *I never change it. I still love it. I love everything (P04).* Lastly, we included photo-elicited interview questions. Photos were produced by participants with IDDs, making them more involved in the research process [34]. Participants seemed to enjoy talking about their photos. For example, participant one was quick to share a photo of themselves walking with their instructor, describe the photo, and provide details about walking and how the instructor made exercise feel easy. At the beginning of that same interview session, the interviewer asked participant one if they wanted to share anything about their walk, and they said *no (P01)*. The photos often acted as starting points for conversations that participants wanted to share and chose to share. Without the photos, we would have missed opportunities to gather data on those experiences from adults with IDDs. In addition to implementing the above practices during interviews, field observations also supported participant-reported perspectives and experiences. Future researchers interviewing individuals with IDDs should consider using these strategies to support gathering accurate, richer data. As we enable the voices of individuals with IDDs so they can be heard and their stories can be told, researchers also have an obligation to recognize signs of social desirability, determine when participants need more information or different contexts to understand a question, and avoid suggestive or leading language or behaviors [35].

### 4.3. Limitations and Future Research

All participants were recruited from the same fitness program and had the same fitness program instructor during their time in the program. This limits the scope of experiences participants had, and it is possible the similarities between participants are rooted in a similarity of experience. Secondly, we did not conduct additional assessments to confirm IDD diagnosis. It is possible participants were assessed with different instruments since multiple instruments were accepted by the postsecondary program to determine eligibility for their program. Future research should explore the experiences of adults with IDDs across various fitness programs as well as interview fitness instructors to gather their experiences working with adults with IDDs. Gathering these experiences could help identify barriers, identify what is working, and lead to the development of higher-quality fitness programs for individuals with IDDs. Future research should also consider examining participant fitness, social, and mental health outcomes along with participant experiences (e.g., a mixed methodology) to determine how experiences are related to outcomes.

## 5. Conclusions

We recognize adults with IDDs experience a range of factors that influence exercise behavior. This study, however, offers firsthand insight on how capabilities, opportunities, and motivation may influence one another in a community fitness program context for adults with IDDs. This study also described and followed which best practices for interviewing individuals with IDDs were implemented and included a discussion on their potential impacts on findings. While these practices are not new (e.g., photo-elicited interview questions), discussing our experiences with these methods can help the field continue to improve methodological practices, particularly when working with individuals whom require more extensive support or accommodation.

## Figures and Tables

**Table 1:** Participant characteristics.

Participant	Age	Identified Gender	Identified Race/Ethnicity	Diagnosis	Reported OHC	Reported Motor Skills	Reported Exercise Days/Week	Years Enrolled in Fitness Class
01	32	Female	Hispanic	ID	None	Poor	2	2 years
02	34	Female	Hispanic	ID	None	Poor	5	4 years
03	50	Male	Hispanic	ID, CP	None	Poor	2	3 years
04	33	Male	White	ID, DS	None	Poor	1	2 years
05	40	Female	White	ID, DS	Seizures	Poor	3–4	3 years
06	43	Male	White	ID, ADHD	Diabetes, hbp	Poor	2	5 years
07	29	Female	White	ID, CP	hc	Poor	1	1 year
08	34	Male	White	ID, DS	None	Poor	2	2 years
09	31	Female	Hispanic	ID, CP	None	Poor	1	3 years
10	27	Female	White	ID, DS	Diabetes	Poor	1	1 year
11	33	Male	White	ID, ADHD	None	Poor	2	1 year

Note. Age based on date of semi-structured interview. Additional gender (e.g., non-binary) and race/ethnicity (e.g., Asian) options were available but not selected. OHC = other health condition, ID = intellectual disability, CP = cerebral palsy, DS = Down syndrome, ADHD = attention deficit hyperactivity disorder, hbp = high blood pressure, and hc = high cholesterol. Motor skills and exercise days/per week are self-reported.

**Table 2:** Interview questions related to COM-B constructs.

COM-B Construct	COM-B Component	Interview Question Example
Capability	Physical	What helps you do a good job in fitness classes? What exercises can you do by yourself? What exercises do you need some help with? If you need help, tell me what that help looks like.
	Psychological	Why do you take fitness classes? How many times per week do you take fitness classes? How many times per week do you exercise in other places?
Opportunity	Physical	How long have you been taking fitness classes? What are some things that help you come to fitness classes? What are some things that stop you from coming to fitness classes? How do you get to fitness class?
	Social	Who supports you during fitness classes? How do instructors support you during fitness classes? How do peers support you during fitness classes? Do people support you with exercise when you are not at fitness class? If yes, who supports you and what do they do to support you?
Motivation	Reflexive	Which exercises do you enjoy the most? Tell me about a time something was hard in fitness class. Tell me about a time something was easy in fitness class.
	Automatic	Are there any incentives for you to go to fitness classes? How do these fitness classes make you feel?

Note. Additional research questions were part of the interview protocol. Not all questions were directly tied to the COM-B constructs.

**Table 3:** Illustrative examples related to capability, opportunity, and motivation in fitness classes for adults with IDDs.

COM-B Component	COM-B Component Description	Themes Identified	Field Observation or Interview Example	Illustrative Quotes
Physical capability	Physical skill, strength, stamina, and balance	Facilitators: instructor support and modifications Barrier: limited coordination/balance	I: Participants described their favorite exercises, exercises that were difficult, and people who help them with exercise.	*P01: TRX is easy for me, help me do squats and stuff. You hold a thing and squat.* *P02: Balancing is hard…nervous about falling.* *P03: Burpees… they are complicated, hard.* *P06: I’m good at swimming…not lifting weights.*
Psychological capability	Knowledge or psychological skills (e.g., understanding and memory)	Facilitators: instructor support and client support Barrier: articulation of health knowledge	FO: After exercise descriptions and demonstrations, the fitness instructor checked for understanding. Participants shook their heads or verbalized they understood or asked for further clarification.	*Instructor: Show me your push up position.* *P02: Like this?* *Instructor: Yes, that looks good!*
Physical opportunity	Time, finances, materials, and resources related to environmental system	Facilitators: instructor support, facility support, and family support Barriers: resource dependence	I: Participants described opportunities they had to exercise across different settings, their preferences, reliance on others to attend classes, and desires to attend the fitness classes more.	*P01: I go walk all the time… in my neighborhood…the park. I do yoga. I like walking more.* *P04: I wish I could go twice a day.* *P09: Here [fitness center] is my favorite place…hang out with friend.* *P11: My dad has to bring me here. I can’t drive.*
Social opportunity	Interpersonal influences, culture, and social norms	Facilitators: instructor support, client support, family support, and community support	FO: Participants observed splitting into partners or small group for exercises. Participants often had side conversations while completing exercises.	*P06: Did you watch the game last night?* *P04: Haha. It was embarrassing.* *P06: Yeah. Um, we better win next week.* *P04: They don’t play next week.* *P06: Oh yeah!*
Reflective motivation	Conscious plans and evaluations (beliefs about what is good and bad)	Facilitators: instructor support, client support, family support	FO: Participant choice was incorporated regularly into fitness sessions. Participant choice in music selection was regularly observed.	*Instructor: Let’s write down everyone’s activity choice for today.* *P06: I want baseball today.* *P08: Let’s do the obstacle course.* *Instructor: What are we listening to today?* *P04: NAS! (Old Town Road)*
Automatic motivation	Emotional reactions, desires, drive, and impulses	Facilitators: preferences for active activities	I: Participants described feelings around exercise and being at the fitness center.	*P03: I exercise and I’m happy.* *P04: [name of fitness center] is my favorite place.* *P10: I have fun.*

Note. I = interview example, FO = field observation example, and TRX = total body resistance exercise.

## Data Availability

The data presented in this study are available upon request from the corresponding author. The data are not publicly available due to participant privacy.
